# Divergent interpretations of child abuse in legal judgments: perspectives from clinicians and forensic experts

**DOI:** 10.1186/s13690-024-01425-y

**Published:** 2024-10-25

**Authors:** Ching-Min Tang, Chen-Fang Lou, Shao-Hsuan Hsia, Kuang-Tsung Liang, Wen Chang, Jainn-Jim Lin, Oi-Wa Chan, Kuang-Lin Lin, En-Pei Lee

**Affiliations:** 1 Division of Pediatric Critical Care Center, Department of Pediatrics, Chang Gung Memorial Hospital, Chang Gung University, 5 Fu-Shin Street, Kwei-Shan, Taoyuan, 333 Taiwan; 2 College of Medicine, Chang Gung University, Taoyuan, Taiwan; 3 Division of Pediatric Neurology, Department of Pediatrics, Chang Gung Memorial Hospital, Chang Gung University, Taoyuan, Taiwan; 4 School of Nursing, Chang Gung University of Science and Technology, Taoyuan, Taiwan; 5 Department of International and Cross-Strait Legal Affairs, Minister of Justice, Taipei, Taiwan

**Keywords:** Child abuse, Abusive head trauma, Forensics, Judgment, Taiwan

## Abstract

**Background:**

Child abuse in Taiwan is a major societal concern that severely affects the well-being of children. Despite the complexity in detecting abuse, reports of child abuse are increasing, evidenced by a rise in cases and heightened awareness. This study utilizes judicial judgments as a lens to understand the varied interpretations of child abuse by clinical and forensic experts and explores the broader epidemiological trends of such abuse within the declining youth population of Taiwan.

**Methods:**

We conducted a retrospective study by analyzing official court judgments on child abuse allegations judged from 2008 to 2022 from the online database of Judicial Yuan. Furthermore, the study analyzed demographic factors, injury patterns, and opinions from various experts.

**Results:**

The results reveal that severe criminal cases of child abuse predominantly involve biological fathers as the primary offenders and physical abuse as the most common form of maltreatment. Victims are typically aged less than 5 years, which frequently leads to an unfavorable prognosis. Analysis also highlights the TEN-4-FACESp acronym as a highly predictive indicator of child abuse and underscores the prevalence of abusive head trauma (AHT). Moreover, the findings emphasize ongoing disparities in opinions between forensic medical examiners and clinical physicians, especially in AHT cases, which potentially influences judicial decisions.

**Conclusions:**

In summary, the study reveals ongoing disagreements between forensic medical examiners and clinical physicians, especially in cases of AHT, which may impact judicial decisions. Promoting consensus through interdisciplinary collaboration and improved communication can aid in revealing the truth in child abuse cases.

**Table:** 

**Text box 1. Contributions to the literature**
• This study provides a unique perspective on child abuse in Taiwan by analyzing court judgments, offering insights into the legal interpretation of abuse cases.
• It highlights the ongoing disagreement between forensic and clinical experts in child abuse cases, especially in abusive head trauma (AHT), emphasizing the need for better interdisciplinary communication.
• The research confirms the effectiveness of the TEN-4-FACESp tool in identifying potential child abuse cases, supporting its wider adoption in clinical practice.
• By examining child abuse trends in Taiwan’s declining youth population, this study contributes to the understanding of changing patterns in child maltreatment within a specific demographic context.

## Introduction

Child abuse, which pertains to various forms, including physical, emotional, and sexual abuse and neglect, constitutes an alarming societal concern that exerts detrimental impacts on the well-being of children [1]. The detection of child abuse proves challenging, primarily because children typically lack the ability to provide accurate descriptions, particularly of cases without witnesses. Among instances of physical abuse, abusive head trauma (AHT) poses the most complex challenge in terms of diagnosis and judgment, because evident injuries, such as bruises, may be lacking. AHT stands as a global health crisis, representing the foremost cause of infant morbidity and mortality worldwide. As the most severe form of child abuse, AHT carries devastating consequences, with a mortality rate of 20% and long-term neurological impairment rates reaching up to 50% [2]. A population-based study in the United States revealed an incidence rate of approximately 30 cases per 100,000 person-years in children [3]. In Taiwan, AHT emerged as the predominant form of injury among abused children, particularly affecting infants under one year of age. This finding was based on a comprehensive study of 1,212 hospitalized abuse victims between 1997 and 2009 [4].

Taiwan’s population stands at 23,415,100 as of April 2024, with child and adolescent numbers expected to drop from 3.42 million in 2022 to between 1.19 and 2.1 million by 2070. While the youth demographic declines, child protection cases have risen, with reports of abuse occurring every 1.09 h last year and a 10% increase in cases in the first quarter compared to the previous year [5]. This trend suggests not only increased reporting of abuse but also a concerning rise in actual incidents, highlighting the need for enhanced child welfare measures.

To fully comprehend the intricate details of such issues, we examined judgments related to child abuse as the primary material for analysis. The importance of a judgment stems from its role as a formal decision rendered by a judge in a legal case. As the ultimate outcome of a trial, judgment plays a pivotal role in determining the legal consequences of the case. Judicial decisions in suspected AHT cases frequently rely on expert testimony from clinicians and forensic doctors. However, extant literature demonstrates significant discrepancies between these experts in their interpretation of injury etiology, particularly regarding SDH [6]. This inter-expert variability poses substantial challenges for legal adjudication in such cases. In this context, the significance of the study lies in its use of judgments as crucial materials and analysis of the expert opinions of clinical physicians, court-appointed physicians, and forensic medical examiners that underlie judgments. By dissecting and comparing final legal decisions and viewpoints, the study intends to elucidate discrepancies among the opinions of these experts and their potential impact on judgment outcomes. Additionally, the study investigates the epidemiology and characteristics of child abuse in Taiwan.

## Materials and methods

A retrospective study was conducted using official court judgments of child abuse allegations from 2008 to 2022. All official documents are stored in the public online database of Judicial Yuan, which is one of the five branches of the government of Taiwan and serves as the highest judicial organ in Taiwan’s legal system. The official documents covered statements from clinical physicians, court-appointed physicians, and forensic medical examiners. If a defendant does not contest the admissibility of evidence, then the diagnosis of a clinical physician may be admitted as evidence in court for consideration without the need for oral testimony. Court-appointed physicians, similar to expert witnesses, were engaged as second-opinion providers when the judge required the clarification of the clinical course of the victim. Additionally, in cases in which the victim does not succumb to injuries, then the opinions of forensic medical examiners may not be sought. Critically evaluate and compare the expert opinions, focusing on key discrepancies and their potential influence on the judicial decision. To ensure privacy and confidentiality, the study meticulously anonymized the data to prevent any identification of individual children. The study excluded cases involving hired caregivers and sexual abuse.

In Taiwan, if a healthcare worker suspects child abuse, they must first notify the social worker from the Social Affairs Bureau. The social worker will provide the prosecutor with information based on the available medical evidence. The prosecutor will then decide whether to proceed to court based on this evidence.

## Results

### Case selection

**Fig. 1:**
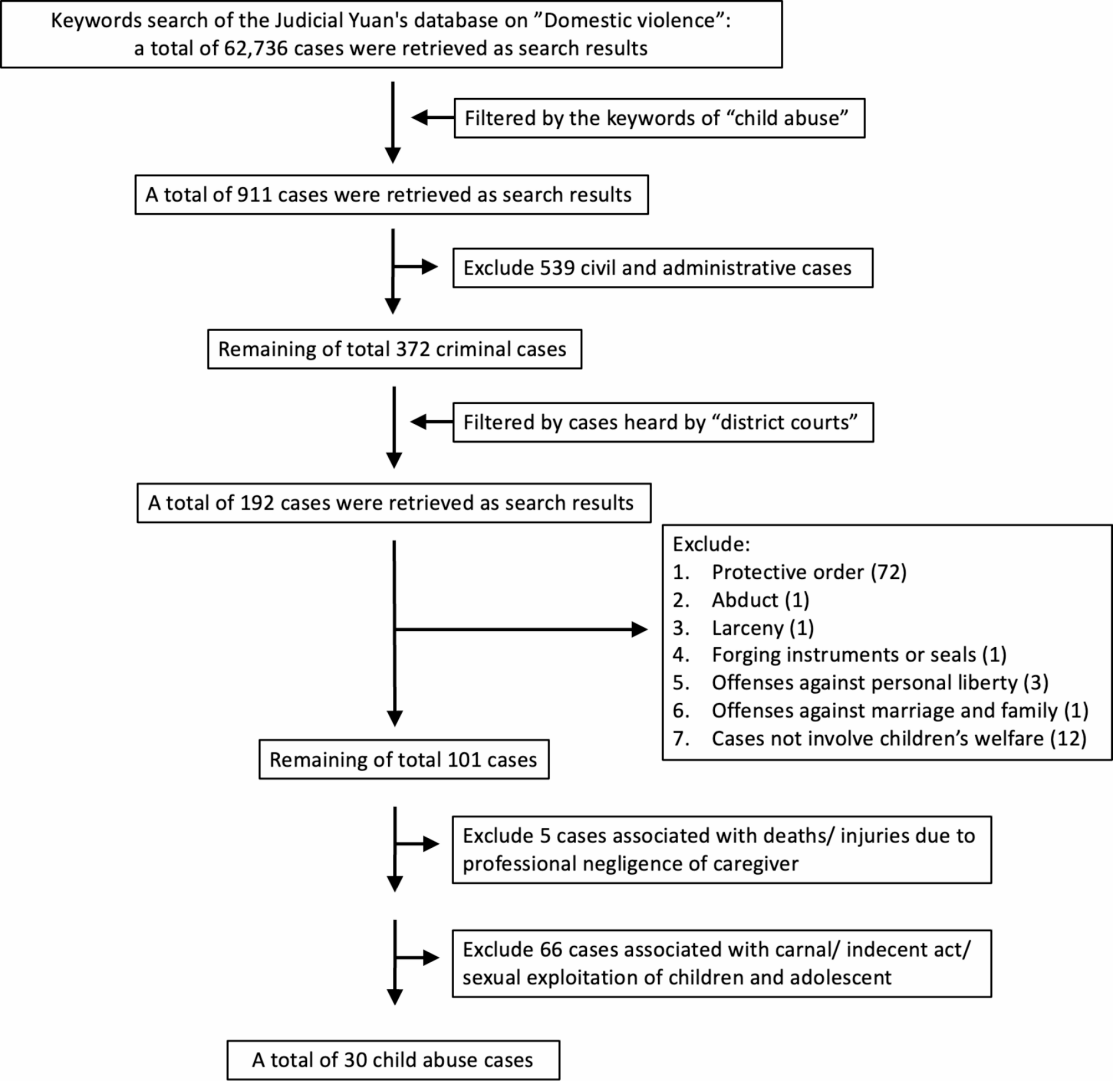
Flow chart of the study From 2008 to 2022 in Taiwan, initial search identified 62,736 domestic violence cases, which expanded to 911 upon inclusion of “child abuse” keyword. Following exclusions, 372 cases remained, of which 192 were from district courts. Further refinement removed cases with civil protection orders (72), abduction, (1) larceny, (1) forging, (1) personal freedom offenses, (3) and marriage/family offenses, (1) along with 12 unrelated to children’s welfare. This resulted in a final selection of 101 cases. Subsequent exclusions of hired caregiver (5) and sexual abuse (66) cases yielded a total of 30 for analysis

To retrieve the relevant cases from the database of Judicial Yuan, we conducted a comprehensive keyword search from 2008 to 2022 (Fig. 1). The initial search yielded 62,736 cases related to domestic violence. Further filtering was conducted by incorporating the keyword “child abuse,” which resulted in 911 cases. Subsequent exclusions were made based on case types, which led to the omission of 539 civil and administrative cases. The remaining 372 cases were then filtered by cases heard by district courts, which produced a subset of 192 cases. Additional exclusion criteria were applied, thus excluding cases including civil protection orders (72), forcible abduction [1], larceny [1], forging instruments or seals [1], offenses against personal freedom [3], and offenses against marriage and family [1]. Cases unrelated to children’s welfare [11] were also excluded. The refinement process led to a final selection of 101 cases. Finally, cases involving hired caregivers [5] and sexual abuse (66) were excluded. Ultimately, a total of 30 cases were included in the analysis.

### Demographic factors

Table 1 presents the results of analysis of demographic factors in relation to child abuse after a specific examination of the types of maltreatment, perpetrators, age range of victims, and prognoses. Among the various types of maltreatment, physical abuse (50%) emerged as the most prevalent form followed by physical and emotional abuse (30%). In terms of perpetrators, the study identified the biological father as the primary offender (30%) followed by the biological mother (27.6%). The majority of victims fell within the age range of 1 to 5 years (56.7%). Furthermore, the prognosis for these victims was predominantly severe with outcomes ranging from death (56.7%) to a severe disability or a vegetative state (26.7%). This result highlights their vulnerability and emphasizes the critical nature of child abuse, which demands increased attention.

**Table 1:** Demographics of child abuse about the types of maltreatment, perpetrators, age range of victims, and prognoses from 2008 to 2022 in Taiwan

	Cases	Percentage
Types of maltreatment		
Neglect only	3	10%
Physical abuse only	15	50%
Physical abuse and neglect	3	10%
Physical and emotional abuse	9	30%
Perpetrators of physical abuse of children		
Biological mother	8	27.6%
Biological father	9	30%
Stepmother	1	3.3%
Stepfather	4	13.3%
Adoptive parents	1	3.3%
Other relatives	5	16.7%
Unknown	2	6.7%
Age of victims (years)		
< 1	9	30%
1–5	17	56.7%
6–12	2	6.7%
13–18	2	6.7%
Prognosis of victims (PCPC^A^ score)		
Normal	1	3.3%
Mild disability	2	6.7%
Moderate disability	2	6.7%
Severe disability/vegetative state	8	26.7%
Death	17	56.7%

^A^PCPC: Pediatric Cerebral Performance Category

### Injury patterns

Table 2 presents an in-depth analysis of injury patterns and related conditions observed in the 30 judgments. The primary pattern identified as the most relevant was that of TEN-4-FACESp (83.3%), which pertains to bruises in the trunk (T), ears (E), and neck (N) in children under the age of 4 years or any bruise in children less than 4 months old [4], facial injuries, including the frenulum (F), angle of jaw (A), cheeks (C), eyelids (E), subconjunctivae (S), and patterned brushing (P) [7]. The second most prevalent finding (80%) was the coexistence of multiple abuse patterns alongside the primary one. This encompassed a spectrum of abusive injuries coupled with indicators of neglect, including malnutrition, failure to thrive, poor hygiene, inadequate clothing, and untreated medical conditions. Additionally, emotional abuse manifestations, such as withdrawal, anxiety, or developmental delays, were often observed concurrently. This finding suggested an association between physical abuse and neglectful behaviors, which potentially exacerbates the overall harm inflicted on victims. Intracranial injuries (63.3%), such as subdural hemorrhage (SDH), subarachnoid hemorrhage (SAH), or intracranial hemorrhage, rank as the third most frequent injury pattern. Another prominent pattern involves fractures (53.3%), which highlight the vulnerability of this age group to physical abuse. These fractures can occur in various body parts and are significant indicators of abuse. AHT (36.7%) accounts for more than half of intracranial injuries, which underscores its prevalence in child abuse cases. However, factors, such as cigarette burns (4%) and oral injuries (4%), contribute to a smaller percentage of the overall results.

**Table 2:** Analysis of injury patterns observed in the 30 cases from 2008 to 2022 in Taiwan

	Total cases (%)
TEN-4-FACESp	25 (83.3%)
Presence of other abusive signs	24 (80%)
Intracranial injuries	19 (63.3%)
Fractures	16 (53.3%)
Abusive head trauma	11 (36.7%)
Cigarette burns	4 (13.3%)
Injuries to mouth	4 (13.3%)

### Variations in expert opinions

A comparative analysis of expert decisions revealed variations in assessments and opinions among experts (Table 3). Out the 27 guilty judgments, clinical physicians reported 18 as child abuse cases (67%), while their opinions were not mentioned for the remaining 9 (33%). Strikingly, for the three cases with a judgment of not guilty, clinical physicians deemed that all of them were child abuse cases (100%). In the case of court-appointed physicians, they considered 13 out of the 27 judgments of guilty as child abuse cases (48%), while their opinions were absent for the other 14 (52%). Regarding cases with a judgment of not guilty, court-appointed physicians perceived them as cases of child abuse (100%). Finally, forensic medical examiners confirmed 15 of the guilty judgments as guilty (56%), while one guilty judgment was reversed to ‘not guilty’ (4%), and 11 judgments were not assessed by them (41%). Among the three cases with a judgment of not guilty, forensic medical examiners considered one as not guilty (33%), while their opinions were not mentioned for the two other cases (67%).

**Table 3:** Comparison of judgment decisions by different experts

Judgment results (*n*; %)	Guilty (27; 90%)			Not Guilty (3; 10%)		
Experts’ opinions	F.A^A^	N.A^B^	N.M^C^	F.A^A^	N.A^B^	N.M^C^
Clinical physician (n)	18	0	9	3	0	0
Court-appointed physician (n)	13	0	14	3	0	0
Forensics (n)	15	1	11	0	1	2

^A^FA: Favor abuse; ^B^N.A: not abuse; ^C^N.M: not mention

### The discrepancy judgment between different experts

Table 4 delineates four cases exhibiting significant inter-expert discrepancies. It encompasses three acquitted cases, elucidating the rationale behind each acquittal, and one conviction where forensic medical examiners diverged from the guilty verdict. The first two cases involve victims that exhibited signs of AHT. However, the court declared both cases as not guilty because the evidence to conclusively identify a perpetrator, including any potential witnesses, was insufficient. Furthermore, due to the uncertain timeline implied by the available medical evidence, a judgment of not guilty was reached, even in the presence of a definitive AHT diagnosis.

**Table 4:** The notable discrepancy judgment between different experts

	Final legal decision	Clinical physicians	Court-appointed physicians	Forensic medical examiners	Case details	Reasons for acquittal
Case 1	Not Guilty	Guilty	Guilty	Not mentioned	1-year-old, AHT with old and new SDH, bilateral retinal hemorrhage	Lack of convincing evidence and unclear perpetrator timeline.
Case 2	Not Guilty	Guilty	Guilty	Not mentioned	8-month-old, AHT with old and new SDH, bilateral retinal hemorrhage	Lack of convincing evidence and unclear perpetrator timeline.
Case 3	Not Guilty	Guilty	Guilty	Not guilty	8-month-old, prior hypoxic brain injury, multiple fractures involving ribs and growth plates	The forensic medical examiner deduced that the metaphyseal fractures were attributed to unintentional injury while assisting the patient during rehabilitation.
Case 4	Guilty	Guilty	Guilty	Not guilty	11-month-old, AHT with old and new SDH, bilateral retinal hemorrhage	-

In the third case, a one-year-old child who experienced hypoxic brain injury briefly after birth presented with evidence of multiple fractures involving metaphyseal and old and new SDH. Although the clinical and court-appointed physicians concluded that the injuries were consistent with child abuse, the forensic medical examiners determined a non-abusive cause due to the child’s prolonged bedridden conditions, which rendered the child susceptible to fracture and bleeding, determining the case to be non-abusive. The forensic medical examiner inferred that the metaphyseal was the result of an accidental injury during the rehabilitation of the patient. Furthermore, the presence of old and new SDH could be associated with the abovementioned susceptibility to bleeding.

In light of the prior history of the child of choking and the resultant hypoxic ischemic brain injury, the clinical and court-appointed physicians harbored significant suspicions regarding the possibility of a high-risk child abuse scenario. Upon revisiting the history of the case, retinal hemorrhage was initially diagnosed. Nevertheless, the analysis of forensic medical examiners suggested that the retinal hemorrhage may have been linked to prolonged hypoxia events and associated acidosis, which led to an increased tendency of bleeding.

The fourth case involved an 11-month-old female infant, ultimately judged guilty of AHT inflicted by her biological mother. The victim succumbed to neurogenic shock and recurrent sepsis two weeks post-admission to the intensive care unit. Notably, the forensic medical examiners faced challenges in identifying AHT evidence after this prolonged interval, leading them to conclude a non-abusive etiology.

These examples underscore the inherent complexity and challenge associated with the identification and prosecution of child abuse cases, particularly in instances involving AHT. It also reveals discrepancies among the opinions of various experts. The forensic medical examiners’ hypothesis—that spontaneous SDH can occur in bedridden children and that RH may result from hypoxia—appears inconsistent with current evidence [8].

## Discussion

Child abuse, which is a pervasive issue worldwide, casts a glaring spotlight on the crucial topic of child welfare [1]. With its complex nature, AHT places an additional layer of intricacy to the equation. AHT is characterized by a diagnostic triad that consists of intracranial SDH, cerebral edema with hypoxic–ischemic changes, and retinal hemorrhage [9]. The etiology of the injury is predominantly multifactorial, involving various factors including rotational acceleration-deceleration such as shaking, impact, or both. However, a debate is ongoing that these symptoms may stem from accidental causes instead of abuse given the potential lack of conclusive evidence to firmly establish abuse as the cause [10, 11]. Additionally, a recognition emerges that benign external hydrocephalus with subdural hematoma may be mistakenly diagnosed as AHT [12], and retinal hemorrhage may be linked to increased intracranial pressure, meningitis [13], or accidental injuries.

Reaching a conclusive determination in cases of AHT is not a straightforward task, because it demands a careful examination of multifaceted evidence and expert perspectives. By facilitating collaboration among experts with diverse opinions, a comprehensive and accurate understanding of the truth can ultimately be attained. To the best of our knowledge, this study is the first to analyze the opinions of clinical physicians, court-appointed physicians, and forensic medical examiners based on official court judgments on child abuse allegations.

Based on the findings, the study observes a notable discrepancy between the viewpoints of forensic medical examiners and clinical physicians. In the abovementioned case, the conclusions drawn by the forensic medical examiners contradicted the opinions of the clinical and court-appointed physicians. This conflict led to the case being concluded with a judgment of not guilty. The forensic medical examiners inferred the metaphyseal fracture as an unintentional injury during the rehabilitation process. Nonetheless, highlighting that metaphyseal fractures are typically indicative of a high specificity for child abuse is important [14–16].

Understanding the characteristics of retinal hemorrhage is crucial for a differential diagnosis. In cases of child abuse, retinal hemorrhage is characterized by multilayered bleeding in the retina as a result of repetitive acceleration–deceleration shearing forces in the blood vessels. Additionally, severe hemorrhagic retinopathy is uncommon even in severe cases of coagulopathies [17]. Among individuals with nontraumatic illnesses and coagulopathies, small punctate retinal hemorrhages may be visible. Importantly, these hemorrhages differ from those observed in victims of inflicted head trauma [18]. Forensic reports lacked detailed descriptions of retinal hemorrhage morphology, potentially impacting diagnostic and legal conclusions. Therefore, providing a comprehensive description that encompasses the quantity, type, and distribution pattern of hemorrhages is of utmost importance for distinguishing cases of child abuse.

Moreover, providing a chronological assessment of the age of SDH could offer valuable assistance to the judge in establishing the potential perpetrator. In the instances of acquittal outlined in the study, the absence of corroborative evidence led to a judgment of not guilty despite the evident severe consequences suffered by the victims as a result of AHT. In this context, the role of a clinical physician involves the utilization of available medical evidence to establish a time frame. For example, the physician could potentially determine the approximate timing of the incident by examining the visible characteristics of the hematoma during surgery [19]. Equipped with this temporal insight, the judge can then make informed inferences regarding the probable perpetrator. Temporal clinical manifestations may differentiate between cases of repeated maltreatment and instances of spontaneous hemorrhage recurrence [20].

The disparity among various experts requires increased scholarly attention. Such a divergence can be attributed to the fundamental differences in the objectives of these two evaluations, which consequently yield distinct outcomes [21]. For instance, the assessment of a clinical physician is intended to determine the etiology and offer an optimal treatment to alleviate discomfort, whereas the evaluation of a forensic medical examiner serves the purpose of addressing legal inquiries. Moreover, the opinions provided by the majority of forensic medical examiners are inferred from on the basis of the results of examining and dissecting the body. Alternatively, clinical physicians typically conduct comprehensive assessments within the clinical setting. Thus, discrepancies in results may exist between the two experts due to fundamental differences in their objectives and evaluation processes. Judicial processes, including cross-examination and evidentiary standards, should function to scrutinize and validate expert testimonies. In addition, to address the discrepancies between clinical and forensic outcomes related to child abuse in Taiwan, several steps should be taken: Establish a multidisciplinary expert panel comprising representatives from medical, forensic, social work, and legal fields. This panel would convene to discuss and review controversial cases. Enhance knowledge and awareness of child abuse, particularly AHT, among legal professionals and frontline workers, including social workers. Develop a centralized, comprehensive database to collect and analyze data related to child abuse cases. This would facilitate case analysis, pattern identification, and inform future diagnoses and judicial decisions. Additionally, this database could serve as a platform to improve communication channels between various stakeholders involved in child abuse cases. These recommendations are not only applicable to Taiwan but could also be implemented globally to reduce discrepancies and improve outcomes in child abuse cases. The approach of combining expert consultation, education, and integrated data analysis and communication can be adapted to different cultural and legal contexts worldwide.

## Limitations

An inherent limitation of the study is the relatively small sample size. Despite conducting a judgment search across a 14-year period, the deliberate exclusion of child abuse cases involving sexual abuse and those related to babysitters, which is intended to maintain the focus of the study, holds the potential to impact the overall study population. Consequently, this limitation may influence the generalizability and comprehensiveness of the findings.

## Conclusion

The study identified key characteristics of child abuse in Taiwan, including the predominant role of biological fathers as perpetrators, physical abuse as the primary form of maltreatment, and victims primarily aged between 1 and 5 years with a generally poor prognosis that typically results in death. Additionally, the findings underscore some disparities in opinions between forensic medical examiners and clinical physicians, especially in cases of AHT, which may impact judicial decisions. To foster consensus, integrating viewpoints from various experts and promoting enhanced communication can contribute to revealing the truth that underlies child abuse.

## Data Availability

No datasets were generated or analysed during the current study.

## Abbreviations

AHT: Abusive head trauma
PCPC: Pediatric Cerebral Performance Category
SAH: Subarachnoid hemorrhage
SDH: Subdural hemorrhage
TEN-4-FACESp: trunk (T), ears (E), and neck (N) in children under the age of 4 years or any bruise in children less than 4 months old (4), facial injuries (F), angle of jaw (A), cheeks (C), eyelids (E), subconjunctivae (S), and patterned brushing (P)
